# Diagnostic accuracy of magnetic resonance, computed tomography and contrast enhanced ultrasound in radiological multimodality assessment of peribiliary liver metastases

**DOI:** 10.1371/journal.pone.0179951

**Published:** 2017-06-20

**Authors:** Vincenza Granata, Roberta Fusco, Orlando Catalano, Antonio Avallone, Raffaele Palaia, Gerardo Botti, Fabiana Tatangelo, Francesco Granata, Marco Cascella, Francesco Izzo, Antonella Petrillo

**Affiliations:** 1 Division of Radiology, “Istituto Nazionale Tumori - IRCCS - Fondazione G. Pascale”, Naples, Italy; 2 Division of Abdominal Oncology, “Istituto Nazionale Tumori - IRCCS - Fondazione G. Pascale”, Naples, Italy; 3 Division of Hepatobiliary Surgical Oncology, “Istituto Nazionale Tumori - IRCCS - Fondazione G. Pascale”, Naples, Italy; 4 Division of Diagnostic Pathology, “Istituto Nazionale Tumori - IRCCS - Fondazione G. Pascale”, Naples, Italy; 5 Departement of Civil and Mechanical Engineering, University of Cassino and Southern Lazio, Cassino, Italy; 6 Division of Anesthesia, Endoscopy and Cardiology, “Istituto Nazionale Tumori - IRCCS - Fondazione G. Pascale”, Naples, Italy; Texas A&M University, UNITED STATES

## Abstract

**Purpose:**

We compared diagnostic performance of Magnetic Resonance (MR), Computed Tomography (CT) and Ultrasound (US) with (CEUS) and without contrast medium to identify peribiliary metastasis.

**Methods:**

We identified 35 subjects with histological proven peribiliary metastases who underwent CEUS, CT and MR study. Four radiologists evaluated the presence of peribiliary lesions, using a 4-point confidence scale. Echogenicity, density and T1-Weigthed (T1-W), T2-W and Diffusion Weighted Imaging (DWI) signal intensity as well as the enhancement pattern during contrast studies on CEUS, CT and MR so as hepatobiliary-phase on MRI was assessed.

**Results:**

All lesions were detected by MR. CT detected 8 lesions, while US/CEUS detected one lesion. According to the site of the lesion, respect to the bile duct and hepatic parenchyma: 19 (54.3%) were periductal, 15 (42.8%) were intra-periductal and 1 (2.8%) was periductal-intrahepatic. According to the confidence scale MRI had the best diagnostic performance to assess the lesion. CT obtained lower diagnostic performance. There was no significant difference in MR signal intensity and contrast enhancement among all metastases (p>0.05). There was no significant difference in CT density and contrast enhancement among all metastases (p>0.05).

**Conclusions:**

MRI is the method of choice for biliary tract tumors but it does not allow a correct differential diagnosis among different histological types of metastasis. The presence of biliary tree dilatation without hepatic lesions on CT and US/CEUS study may be an indirect sign of peribiliary metastases and for this reason the patient should be evaluated by MRI.

## Introduction

Peribilary metastases are rare, although are the most common solid malignancy of the bile ducts beyond cholangiocellular carcinoma (CCC) [1]. Cancers most frequently metastasizing to the biliary tract are those of the gastrointestinal tract, such as colorectal, gastric, and pancreatic carcinoma. In addition, breast, lung, and renal cancer, as well as melanoma and lymphoma, can localize to the biliary tract [1]. The site more often involved is represented by the common hepatic duct, as extraluminal mural tumor tissue or as hepatoduodenal ligament lymph-nodes metastasis [1–2]. Proper recognition of metastatic lesions is crucial for patient management. Imaging studies do not allow a differential diagnosis with CCC, and sometimes from non-tumor lesions, due to an overlapping of radiological features [3]. Although PET is a sensitive technique for the detection of peribiliary tumors, it is not specific in their differential diagnosis [3]. Consequently, diagnosis is frequently based on histological findings and on the patient medical history [3]. Multidetector computed tomography (MDCT) and magnetic resonance imaging (MRI) are the most common tools employed to evaluate the liver and biliary tree, playing a critical role in the diagnosis, staging, and treatment planning [4]. Although ultrasound (US) is sometimes the first modality used in the evaluation of biliary tract disease, the accuracy of US varies according to patient body habitus and the operator skill [5]. US is not accurate in the estimation of tumor spread and in the determination of tumor resectability compared to CT or MRI [6–7]. CT and MRI are a comprehensive imaging modality with multiplanar capability to assess the liver parenchyma and biliary tree. MRI provides an assessment of the signal characteristics, vascularity, and pathophysiology of different tumors due to its superior soft-tissue contrast [8–9]. Given that this information is crucial for tumor staging and treatment planning, MRI is the preferred imaging modality for patients with suspected biliary tumors [6]. The purpose of this study was to compare the diagnostic performance of MDCT, US without and with contrast medium (CEUS) and MRI in patients with proven peribiliary metastases..

## Materials and methods

### Patient population

Review board approval by National Cancer Institute of Naples Pascale Foundation was obtained for this retrospective study. Each enrolled patients provided informed consent. Through a computerized search of medical records, 35 oncological patients (Table 1), with an increase of bilirubin (between 4 mg/dl and 16 mg/dl; mean value 8 mg/dl) and Carboihydrate Antigen (CA) 19.9 (between 60 U/ml and 148 U/ml; mean value 109 U/ml) with proven histological peribiliary metastases were identified. All patients have been underwent gadolinium ethoxybenzyl diethylenetriamine pentaacetic acid (Gd-EOB-DTPA)-enhanced MRI, MDCT and US/CEUS studies from May 2012 to December 2016. The radiologists knew the oncological history of patients.

**Table 1:** Patients demographics data.

Description	Numbers (%)
Gender	Men 17 (48.6%)
	Women 18 (51.4%)
Age	58 (range 42–80)
**Concomitant or previous history of**	
Colorectal Adenocarcinoma	12
Breast Cancer	7
Pancreatic Adenocarcinoma	7
Gastric Adenocarcinoma	5
Endometrial Cancer	1
Ovarian Cancer	3
**Concomitant and previous history of liver intraparenchimal metastases**	8 (22.8%) and 19 (54.3%)
**Clinical symptoms**	
Abdominal pain	12 (34.3%)
Jaundice	28 (80.0%)
Pruritus	18 (51.4%)
Increase of tumor markers	35 (100.0%)
Systemic symptoms (Weight loss or anemia)	5 (14.3%)

As a control study groups, we identified 16 consecutive patients with periductal-infiltrating cholangiocellular carcinoma (PI-CCC) (group A) and 30 consecutive patients with intra parenchymal liver metastases by colorectal cancer (group B) without increase of bilirubin and CA 19.9. In Tables 2 and 3 we reported the data about the control groups.

**Table 2:** Control group A demographics data.

Description	Numbers (%)
Gender	Men 5 (31.2%)
	Women 11 (68.7%)
Age	62 (range 54–78)
**Side**	Perihilar (p)-CCC(16; 100.0%), 100.0% type IV according to Bismuth
	Intrahepatic (i)-CCC (0; 0.0%)
Liver cirrhosis	0 (0.0%)
**Clinical symptoms**	
Abdominal pain	8 (50.0%)
Jaundice	16 (100.0%)
Pruritus	13 (81.2%)
Increase of CA 19.9	16 (100–0%)
Systemic symptoms (Weight loss or anemia)	7 (43.7%)

**Table 3:** Control group B demographics data.

Description	Numbers (%)
Gender	Men 19 (63.3%)
	Women 11 (36.6%)
Age	58 (range 40–82)
Side	Right lobe 28 (93.3%)
	Left lobe 17 (56.7%)
Numbers	5 (range 2–8)
**Clinical symptoms**	
Abdominal pain	4 (13.3%)
Jaundice	9 (30.0%)
Pruritus	3 (10.0%)
Increase of CA 19.9	1 (3.3%)
Systemic symptoms (Weight loss or anemia)	17 (56.7%)

The study had a retrospective nature. It was performed in accordance with local ethical committee regulation. Each patients provided the consent for data publication.

### MDCT imaging protocol

Non contrast-enhanced phase and triple-phase contrast-enhanced MDCT was performed with a 64-detector row scanner (Optima 660, GE Healthcare, USA). MDCT scanning parameters were 120 kVp, 100–470 mAs (NI 16.36), 2.5-mm slice thickness and table speed 0.984/1mm/rotation. Scans were carried out including a region encompassing the liver from diaphragm to iliac crests. Phases were as follows; hepatic arterial phase 30–40 s after injection of 120 mL of a nonionic contrast medium (Iomeprol, Iomeron 400, Bracco, Milan, Italy) with a bolus-triggered technique (120 kVp; 40–60 mA), portal and equilibrium phase 90 s and 120 s after contrast injection. The contrast medium was administered at a rate of 4 mL/s through antecubital vein with an automated injector system (Empower CTA, E-Z-EM Inc., New York, USA).

### MR imaging protocol

MR imaging was performed by using a 1.5 T scanner (Magnetom Symphony, with Total Imaging Matrix Package, Siemens, Erlangen, Germany) with an 8-element body coil and a phased array coil. The MR examination consisted of images taken before IV injection of contrast medium and dynamic sequences obtained after injection of a liver-specific contrast medium (Primovist, Bayer Schering Pharma, Berlin, Germany). Our MR protocol is summarized in Table 4. Diffusion weighted imaging (DWI) was obtained with planar echo-pulse sequence [*b* values 0, 50, 100, 200, 400, 600, and 800 s/mm^2^]. All patients received 0.1 mL/kg of Primovist (5–10 mL, mean 8 mL) by means of a power injector (Spectris Solaris^®^ EP MR, MEDRAD Inc., Indianola, USA), at a rate of 1 mL/s. VIBE T1-weighted fat-suppressed (SPAIR) sequences were acquired in four different phases: arterial phase (35 s delay), portal venous phase (90 s), late/transitional phase (120 s) and hepatobiliary excretion phase (20 minutes).

**Table 4:** Pulse sequence parameters on MR studies.

Sequence	Orientation	TR/TE/FA (ms/ms/deg.)	AT (min)	Acquisition Matrix	ST/Gap (mm)	FS
TRUFISP T2-W	Coronal	4.30/2.15/80	0.46	512x512	4 / 0	without
HASTE T2-W	Axial	1500/90/170	0.36	320x320	5 / 0	Without and with (SPAIR)
HASTE T2-W	Coronal	1500/92/170	0.38	320x320	5 / 0	without
In-Out phase T1-W	Axial	160/2.35/70	0.33	256x192	5 / 0	without
DWI	Axial	7500/91/90	7	192x192	3 / 0	without
Vibe T1-W	Axial	4.80/1.76/12	0.18	320x260	3 / 0	with (SPAIR)
3D T2-W	Coronal	4324/702/140	5.16	384x354	1/0	With (SPAIR)

**Note**–TR = Repetition Time, TE = Echo Time, FA = Flip Angle, AT = Acquisition Time, ST = Slice Thickness, FS = Fat suppression, SPAIR = Spectral Adiabatic Inversion Recovery, 3D = three dimensional.

### US and CEUS protocol

CEUS was always preceded by a careful US survey, assessing the size and appearance of the lesion. This baseline assessment was done to appropriately choose the liver area or areas to be particularly focused in the forth- coming contrast-enhanced part of the US study. In all cases, a separated injection was performed for each liver lobe. For both injections, the arterial phase assessment was focused on any known lesion at baseline US. CEUS was performed as a low-mechanical index, double-split mode, real-time modality. We employed a Technos MyLab 70 XVG and MyLab Twice scanner (Esaote, Genoa, Italy), injecting 2.4 ml of a sulfur hexafluoride-based contrast medium (SonoVue, Bracco, Milan, Italy) per each liver lobe. After the injection, the radiologist focused the sonographic field of view on the parenchymal area of interest, waiting for the microbubbles arrival. Thereafter, he/she moved the transducer to explore the remaining parenchyma of each lobe, with special reference to the segment bearing the ablated lesion.

### Images analysis

Four expert hepatic radiologists retrospectively and independently reviewed all images. A consensus read was performed when there was disagreement between the readers. CEUS, CT and MR imaging analysis were done independently at different time. Presence, side, and extent of the lesions on CEUS, CT and MRI study were categorized, using a 4-point confidence scale (score) [10]; 1, no lesion; 2, probably no lesion; 3, probably lesion; 4, definitely lesion. For each single lesion the radiologists recorded the site and extent, the signal intensity (SI) on T1-W images, on T2-W images, on DWI, on the apparent diffusion coefficient (ADC) map of DWI images, the ADC value of lesion, the presence of contrast enhancement during the arterial, portal, equilibrium and hepatobiliary phase of MR study. The radiologists recorded the density and the presence of contrast enhancement during the arterial, portal, and equilibrium phase on CT study. The radiologists recorded the echogenicity on US study and the presence of contrast enhancement during the contrast study on CEUS. The tumor location was classifiedas intraductal, periductal, or intra-periductal, in relation to the site of the lesion, respect to the bile duct. The SI of the lesion on MR sequences was categorized as isointense, hypointense, and hyperintense compared to surrounding liver parenchyma. The density of the lesion on CT study was categorized as isodense, hypodense and hyperdense compared to surrounding liver parenchyma. The echogenicity of the lesion on US and CEUS study was categorized as isoechoic, hypoechoic and hyperechoic compared to surrounding liver parenchyma.

When the lesion was hyperintense on all b values we defined this a restricted diffusion. The DW signal decay was analyzed using a linear fitting of the mono-exponential model, according to the equation ADC = ln (S_0_/S_b_)/b, where S_b_ is the signal intensity with diffusion weighting b (b>200 s/mm^2^) and S_0_ is the non-diffusion-weighted signal intensity. This analysis was based on multiple circular regions of interest (ROIs) of 0.3–0.9 cm^2^ of size using median value of single voxel signals for each *b* value. ROI for the lesion was manually drawn to include such hyperintense voxels on image at *b* value of 800 s/mm^2^. Median diffusion parameter of ROI was used as representative value for each lesion. No motion correction algorithm was used but ROIs were drawn taking care to exclude areas in which movement artifacts or blurring caused voxel misalignments. The data analysis was performed using an in-house software written in Matlab (The MathWorks, Inc., Natick, USA).

We analyzed the enhancement pattern during arterial, portal, and equilibrium phase on CEUS and CT as well as late and hepatobiliary phase on MR study. We recorded if the enhancement was homogeneous, heterogeneous, or progressive. Other aspects evaluated included the presence of biliary tree dilatation, the presence of perilesional changes in vascular perfusion during the arterial phase, the presence of perilesional changes in signal intensity during the hepatobiliary phase, the signal intensity of the biliary ducts during the hepatobiliary phase.

We compared the location, the signal intensity, and the contrast enhancement of PI-CCC (group A) [11–12] compared to metastasis and the peribiliary spaces feature among study population and control study group B (colorectal metastases).

### Statistical analysis

Kruskal-Wallis test was employed to analyze differences in the location, signal intensity, density, echogenicity and contrast enhancement among metastases of different histological type. Kruskal-Wallis test was employed to analyze the differences in ADC median values among metastases of different histotype. Mann Whitney test was employed to analyze the differences between metastases and control group A and control group B. In order to evaluate variability between the readers, inter-reading concordance assessment was assessed by means of Cohen's kappa coefficient (k) for categorical items (0–0.20, poor agreement; 0.21–0.40, fair agreement; 0.41–0.60, moderate agreement; 0.61–0.80, good agreement; and 0.81–1.00, excellent agreement). A *p* value <0.05 was regarded as statistically significant. Statistical analysis was obtained by means of the Statistic Toolbox of Matlab (The MathWorks, Inc., Natick, USA).

## Results

Thirty-five oncological patients were enrolled in this retrospective study. 12 (34,3%) patients had concomitant or previous colorectal adenocarcinoma, 7 (20.0%) ductal breast cancer, 7 (20.0%) pancreatic adenocarcinoma, 5 (14.3%) gastric adenocarcinoma, 1 (2.8%) endometrioid adenocarcinoma and 3 (8.6%) ovarian mucinous cystadenoma. The histological features of metastases reflected those of the primitive cancer.

**Fig 1:**
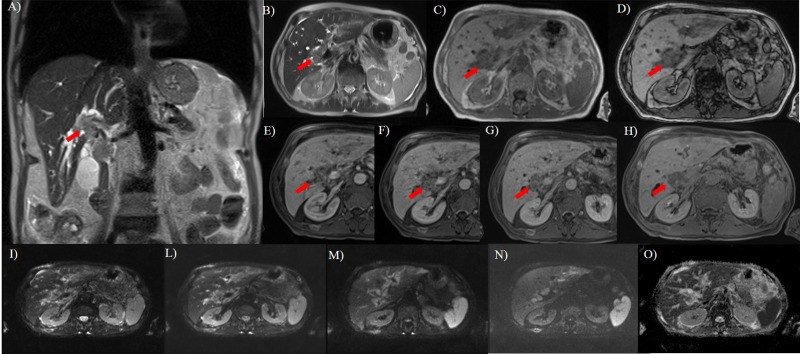
Fifty years old man with rectal cancer. In A (T2-W HASTE coronal plane) and B (T2-W HASTE axial plane), the lesion (arrow) appears as a single periductal hyperintense mass extending to the bifurcations of both right and left hepatic ducts. In C (axial T1-W in phase) and D (axial T1-W out phase) the lesion (arrow) show hypointense signal. During arterial (E), portal (F), equilibrium (G) phase of contrast study the lesion has a progressive contrast enhancement while during hepatobiliary phase (H), is hypointense. DWI sequences: In I *b*0 s/mm^2^, in L *b*50 s/mm^2^, in M *b*400 s/mm^2^, in N *b*800 s/mm^2^ and in O ADC map. The tumor shows restricted diffusion.

**Fig 2:**
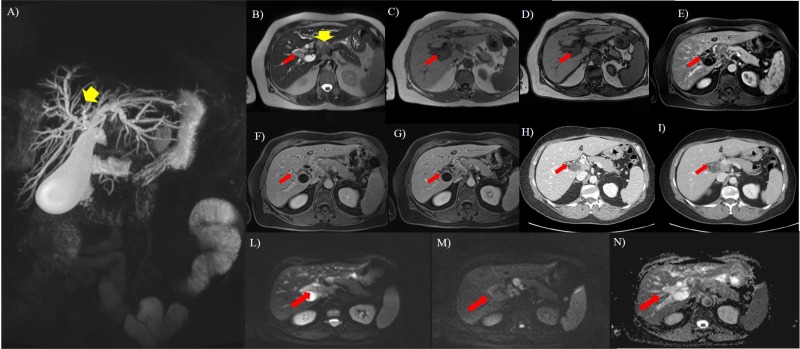
Seventy-three years old woman with breast cancer. A, MIP image. Common hepatic duct is not evident (yellow arrow) and, this is due to a hilar lymph node (yellow arrow) detect in B (axial T2-W HASTE). In b it is also evident the presence (red arrow) of peribiliary tissue as a hyperintense mass extending to the bifurcations of both right and left hepatic ducts. In C (axial T1-W in phase) and D (axial T1-W out phase) the lesion appears as hypointense. After contrast medium (E, arterial, F, portal, G, equilibrium phase) the lesion is not clearly evident so as in arterial phase (H) and portal (I) phase on CT study. DWI sequences: in L *b*50 s/mm^2^, in M *b*800 s/mm^2^, in N ADC map. The tumor (red arrow) shows restricted diffusion.

MR detected all lesions (Fig 1). CT detected the lesion in eight patients (3 patients with colorectal cancer, 2 with pancreatic cancer, 2 with breast cancer, and one with gastric cancer) (Fig 2), while US and CEUS has found one lesion. There was no significant difference in lesion location among all metastases (*p* value >0.05 at Kruskal Wallis test).

According to MR features, the lesions in 34 patients appeared as a single tissue that extended along the biliary tree. In a single patient, with breast cancer, the lesions appeared as multiple individual lesions, and each of them that extended along the bile branches of second order. According to the site of the lesion, respect to the bile duct and hepatic parenchyma,19 (54.3%) lesions were periductal, 15 (42.8%) lesions were intra-periductal and 1 (2.8%) lesion was periductal-intrahepatic (in a patient with endometrial cancer). There was no exclusively intraductal lesion.

US and CEUS detected only the intrahepatic component of a patient with intrahepatic-periductal lesion.

A good concordance among radiologists (k = 0.79 with 95% interval confidence 0.69–0.88) was reported to provide scores. According to the confidence scale the median value obtained, in accordance between all radiologists, was 4 for T2-W, 4 for DWI, 3.6 for T1-W in phase, 3.6 for T1-W out phase, 2 for cholangiographic sequence, 3 for MRI arterial phase, 3.2 for MRI portal phase, 3.2 for MRI late phase, and 3.6 for MRI hepatobiliary phase. For arterial, portal and equilibrium phase the median value was 1.4 on CT study.

**Fig 3:**
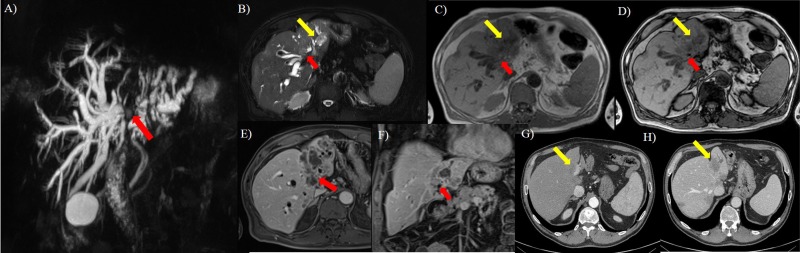
Sixty-three years old man with colorectal cancer. A, MIP image. Interruption of confluence of the right and left hepatic ducts with biliary tree dilation. In B (axial T2-W HASTE FS) it is evident the presence of peri-intraductal ductal hyperintense tissue at confluence of the right and left hepatic ducts (red arrow) and in peri- left hepatic ducts (yellow arrow), that appear hypointense (red and yellow arrow) in C (axial T1-W in phase) and D (axial T1-W out phase), with progressive contrast enhancement in E (arterial phase) and D (portal phase). On CT study in F (arterial phase there is no evidence of lesion, while in portal phase (G) the tumor (yellow arrow) appears as a hypodense hepatic lesion.

**Fig 4:**
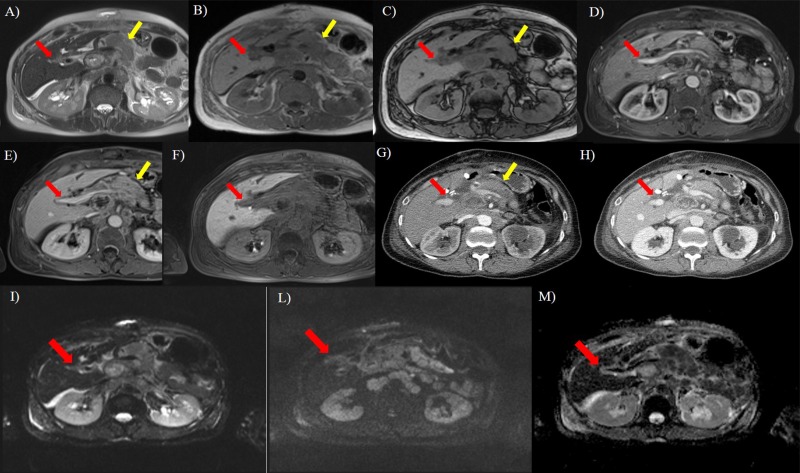
Seventy years old woman with pancreatic cancer (yellow arrow). In A (axial T2-W HASTE) the lesion shows (red arrow) hyperintense signal; in B (axial T1-W in phase) and C (axial T1-W out phase) it appears hypointense (red arrow). During contrast study (D, arterial phase, E, portal phase, F, hepatospecific phase) on MR, there is no evidence of the lesion so as on arterial (G) and portal (H) phase on CT study. DWI sequences: In I *b*50 s/mm^2^, in L *b*800 s/mm^2^, in M ADC map. The tumor (red arrow) shows restricted diffusion.

All lesions showed hypointense signal on T1-W in-out phase images, in pre-contrast T1-W images and in ADC maps, hyperintense signal on T2-W imaging and DWI. The diffusion was restricted from *b*0 s/mm^2^ to *b*800 s/mm^2^ and the median ADC value was of 1.27x10^-3^ mm^2^/s (range, 1.01–1.68x10^-3^ mm^2^/s). All lesions showed a progressive contrast enhancement from arterial to equilibrium phase. All lesions were iso-hypointense in hepatobiliary phase (Fig 3). There was no significant difference in signal intensity and contrast enhancement among all metastases on all imaging acquisitions (*p* value >0.05 at Kruskal Wallis test) (Figs 1–4).

On CT study the detected lesions showed progressive contrast enhancement from arterial to equilibrium phase, with isodense feature on arterial phase, iso-hypodense feature on portal phase and isodense feature on equilibrium phase (Fig 3). There was no significant difference in density and contrast enhancement among all metastases (*p* value >0.05 at Kruskal Wallis test).

In all patients, we found biliary tree dilatation, with a degree of dilatation that correlate with the size of the lesion (Fig 1). When the tumor was of the periductal-intraductal type the degree of dilatation was higher than that for exclusively periductal lesions.

We found no change in perilesion vascular perfusion on hepatic parenchyma during arterial phase of contrast study on CT, MR and CEUS images.

We found no change in perilesion hepatic parenchyma during hepatobiliary-phase on MR study. The biliary ducts adjacent to the metastasis did not eliminate the contrast medium in the hepatobiliary phase on MR study.

All pi-CCC was perihilar (p)-CCC, type IV according to Bismuth, and showed a hypointense signal on T1-W flash in-out phase images and in pre-contrast T1-W images, and a hyperintense signal on T2-W imaging. The diffusion was restricted from *b*0 s/mm^2^ to *b*800 s/mm^2^ and the median ADC value was of 1.46x10^-3^ mm^2^/s (range 1.30–1.57x10^-3^ mm^2^/s). There was no statistically significant difference in median ADC values (*p* value >0.05 at Mann Whitney test). The cholangiocellular lesions showed a progressive contrast enhancement. We found no significant difference in signal and contrast enhancement among all metastases respect to CCC on all imaging acquisitions (*p* value >0.05 at Kruskal Wallis test). The only difference identified was in the extent of disease, with metastases penetrating deeper along the ducts of the second order and CCC showing a minor lesser longitudinal extension. However this difference was not significant (*p* value >0.05 at Mann Whitney test).

In all patients of control group B we found no tissue in periportal/peribiliary space. According to the confidence scale the median value obtained, in accordance between all radiologists, was 0 for T2-W, 0 for DWI, 0 for T1-W in phase, 0 for T1-W out phase, 0 for cholangiographic sequence, 0 for MRI arterial phase, 0 for MRI portal phase, 0 for MRI late phase, and 0 for MRI hepatobiliary phase. For arterial, portal and equilibrium phase the median value was 0 on CT and CEUS study.

## Discussion

Patients with liver metastases may be submitted to different treatments, depending to the extent and location of the lesions [13, 14]. The focus of noninvasive radiological imaging in patients with suspected peribiliary tumors is threefold. First, the tumor must be detected. Second, the lesion has to be appropriately characterized. Third, the resectability must be accurately assessed preoperatively, to avoid ineffective surgery [15]. The presence of peribiliary metastases deeply changes the management of the patient, excluding a radical surgery in cases of lesions involving both biliary system [2–4]. In order to assess tumor etiology and extent as accurately as possible, different imaging modalities are employed jointly in the diagnostic work-up, including CEUS, CT and MRI [15].

Peribiliary metastasis is commonly regarded as extremely rare [1]. We evaluated 35 oncological patients with proven peribiliary metastases from different primitive neoplasm and to the best of our knowledge; this is the first study that analyzes a large group of patients with these lesions. In fact, in our Cancer Center, the percentage of patients with metastases peribiliary was not low. This led us to think that this occurrence may be underestimated. Actually, when we considered the CT evaluation only 22.8% of the cases was properly identified as peribiliary metastasis. CT is usually the first imaging modality employed during cancer patient staging and follow-up. However, our data agree with previous reports showing a limited value of CT in diagnosing biliary system tumors, with a detection rate of 69% and a correct assessment of resectability in 54% [16, 17]. The sensitivity and specificity of CT in differentiating malignant from benign causes of bile duct stricture varies respectively between 82–90% and 65–80% [18]. In our study the diagnostic performance of all CT contrast phases was low (score 1.4). We believe that this depends on the typical progressive contrast enhancement of peribiliary lesions. Consequently, small lesions may go undetected because of the attenuation similar to that of the surrounding parenchyma. For larger lesions, the presence of indirect signs, such as biliary ducts dilatation, may help instead to identify the tumor. We think that the presence of biliary ducts dilatation may suggest the presence of peribiliary tissue and the patient should be evaluated with MRI. In fact, in our series, MRI detected all metastases with the best performance obtained by T2-W and DWI sequences; in fact, according to the confidence scale the median value obtained, in consensus between all radiologists, was 4 for T2-W, 4 for DWI. The high soft tissue contrast resolution in these sequences allows an accurate tumor detection and staging. MRI is the key imaging modality to evaluate liver and biliary tree, also because of the possibility of obtaining cholangiography images [19–23]. The European Society of Gastrointestinal and Abdominal Radiology (ESGAR) Working Group, while providing recommendations on the best use of MRI in the study of liver and biliary tree, suggests the use of DW imaging, perfusion acquisitions, and cholangiopancreatography acquisitions [24]. Our study protocol includes morphological (T1-W and T2-W) and functional (cholangiography sequence, contrast study with a hepatobiliary agent and DWI) sequences, to maximize the tumor detection rate. However, in this study, cholangiography sequence had a lower diagnostic performance compared to T2-W, DWI and contrast study, with a median value obtained, in accordance between all radiologists, of 2. We think that it depends that the lesions are mainly peribiliary and this sequence provides indirect data due to compression. Conversely, we obtained the best performance to detect and to staging the lesion with T2-W and DWI. Park et al. evaluated the incremental benefit of adding DW imaging to gadoxetic acid-enhanced MR imaging and MR cholangiopancreatography in the preoperative evaluation of hilar CCC [20]. As also in our study, DWI improved the assessment of tumor extent along the bile duct. As found also by Choi and coworkers [25], DWI did not improve in our series the diagnostic performance in the characterization of perihilar strictures. In fact, for all metastases and PI-CCC the diffusion was restricted from *b*0 s/mm^2^ to *b*800 s/mm^2^. The median ADC value of metastases was of 1.27x10^-3^ mm^2^/s (range, 1.01–1.68x10^-3^ mm^2^/s) with a data overlapping among lesions. DWI metastases data showed an overlapping with DWI PI-CCC data (median ADC value was of 1.46x10^-3^ mm^2^/s; range 1.30–1.57x10^-3^ mm^2^/s). However, differently from Choi [25], who found that the addition of DWI to morphological MRI was helpful in establishing if bilateral the secondary biliary confluence was involved.

Various liver-specific contrast media have been developed to improve the detection and characterization of the lesion. Gadobenate dimeglumine (Gd-BOPTA) and gadolinium ethoxybenzyl diethylenetriamine pentaacetic acid (Gd-EOB-DTPA) can be injected as an intravenous bolus, providing data about lesion vascularity in the different phases of contrast circulation. Additionally functional data can be obtained in the delayed, hepatobiliary phase [26]. It is known that Gd-EOB-DTPA MRI is the technique to choose to evaluate liver metastases in pre surgical setting [27]. Chung et al. [28] compared the diagnostic accuracy for liver metastases between Gd-EOB-DTPA MRI and DWI, showing that Gd-EOB-DTPA MRI was more useful for the detection of metastases while DWI was more accurate in their characterization. The combination of Gd-EOB-DTPA MRI and DWI had significantly higher accuracy and sensitivity for the preoperative detection of small colorectal liver metastases than DWI alone [28]. However, in our series, the diagnostic performance of post contrast sequences was lower than T2-W and DWI, and it depends of the typical progressive contrast enhancement of the lesion, that appears isointense and the iso-hypointense signal of the lesion on hepatobiliary phase. So that the ratio signal/lesion is lower compared to T2-W sequences.

Although it is known that the histological features of metastases reflected those of the primitive cancer and that intra hepatic colorectal metastases and PI-CCC show a typical MR features [9], in our series, MRI was not helpful in the differential diagnosis between the different metastatic histotypes and metastases respected to CCC, since MR features among all lesions showed an overlapping. There was no significant difference in signal and contrast enhancement among metastases and PI-CCC on all morphological and functional sequences.

US is a modality that has been widely used in past years as a first-line tool in the evaluation of liver metastases. This is because of being inexpensive, noninvasive, and readily available. However, the operator dependence, the ambiguity in side and the necessity of patient’s collaboration reduce the sensitivity and specificity both in detection than in characterization [29–30]. Contrast-enhanced US (CEUS) allows the assessment of dynamic features of liver lesions, increasing the sensitivity and specificity, with an accuracy comparable to CT and MRI, at least in patients adequately accessible to ultrasounds [31]. Several studies have shown that there are not significant differences in diagnostic accuracy between CEUS, CT and MRI to detect liver metastases [31]. However, CEUS had the same limitations as standard US and its sensitivity was dependent on the operator’s skill and patients’ habitus [31]. We found that US and CEUS detected only the intrahepatic component of lesion. No peribiliary lesions were detected by US and CEUS, while US was able to identify biliary tree dilatation in all patients. Therefore, we think that all oncological patients with biliary tree dilatation and without detected lesion on CEUS should be evaluated with MRI to exclude the presence of peribiliary metastases.

## Conclusion

Radiologist should be aware of the US/CEUS, CT and MRI appearance of peribiliary metastasis. This is an uncommon but possible occurrence, probably underrated in its true prevalence. Thanks to its superior soft tissue contrast resolution, the possibility to use contrast media eliminated in the biliary tree, and the possibility to obtain functional data from the DWI, MRI represents an effective tool in the assessment of peribiliary metastasis. MRI was superior to CT and US/CEUS in this setting but it did not allow a correct differential diagnosis between the different hystogical metastases because the overlapping features. The presence of biliary tree dilatation without hepatic lesions on CT and US study may be an indirect sign of peribiliary metastases and for this reason the patient should be evaluated by MRI.
